# The Financial Considerations of Employing a Dedicated Chaperone in Clinical Practice

**DOI:** 10.1177/22925503221128988

**Published:** 2022-10-30

**Authors:** Nick N Maizlin, Stephanie Black, Olubimpe Ayeni

**Affiliations:** 1 Schulich School of Medicine and Dentistry (NNM), Department of Obstetrics and Gynecology (SB), Western University, London, Ontario, Canada; 2 Division of Plastic, Reconstructive & Aesthetic Surgery (OA), 7938 University of Toronto, Toronto, Ontario, Canada

**Keywords:** chaperone, intimate physical examinations, clinical practice, chaperon, examens cliniques intimes, pratique clinique.

## Abstract

**Introduction:** Medical associations and medicolegal bodies are urging for increased chaperone use by physicians during intimate physical examinations in clinical practice (such as breast or pelvic examinations). However, widespread chaperone use is limited by factors such as staff availability and financial considerations. Presently, there is a scarcity of information available regarding the cost of hiring a dedicated chaperone. This study investigates the cost of hiring a chaperone and its financial implications for a physician's clinical practice. **Materials and Methods:** Using data from the Government of Canada website, the range of salary rates for clinic staff who can act as a chaperone in Canada was analyzed. The cost of hiring a chaperone was estimated to be in the range between the cost of hiring a minimum-wage worker and a nurse (the highest-paid hired medical office staff). Obstetrics and Gynecology as well as Plastic Surgery urban community practices were consulted regarding the costs of operating a clinic. **Results:** The approximate annual income for a minimum-wage worker in Canada is $29,250 CAD. Registered nurses earn on average $72,783.75 CAD per year. The cost of operating a private clinic practice with one staff member in Canada is on average $102,500 CAD per year. Thus, hiring an additional full-time chaperone could increase clinic expenses by approximately 49% per year, bringing the clinic cost to approximately $153,517 CAD per year. For part-time employment, the annual cost of hiring a chaperone is approximately $10,203 CAD for each day/week of employment. **Conclusion:** In terms of financial considerations, hiring a chaperone can increase clinic expenses by approximately one-and-a-half times. The findings of this study provide an important reference for physicians and may assist with the decision to employ chaperones in clinical practice.

## Résumé

**Introduction:** Les associations médiales et les institutions médico-légales encouragent fortement le recours à un chaperon lors d’examen physique intime par des médecins en pratique clinique (dans le cas, par exemple, d’examen pelvien ou mammaire). Cependant, le recours généralisé à un chaperon est limité par des facteurs tels que la disponibilité du personnel et des considérations financières. On ne dispose actuellement que de peu d’information sur le coût de l’embauche d’un chaperon dédié. Cette étude porte sur le coût d’un tel recrutement et sur ses implications financières pour la pratique clinique d’un médecin. **Matériels et Méthodes:** Utilisant des données d’un site Web du gouvernement du Canada, nous avons analysé l’éventail des salaires du personnel d’une clinique pouvant servir de chaperon au Canada. Le coût du recrutement d’un chaperon a été évalué comme se situant entre celui d’une embauche au salaire minimum et celui d’une infirmière (l’emploi le mieux payé parmi le personnel médical d’une clinique). Les cliniques communautaires urbaines d’obstétrique et de gynécologie, ainsi que les cliniques de chirurgie plastique ont été consultées pour ce qui concerne les coûts de fonctionnement des cliniques. **Résultats:** Le revenu annuel moyen approximatif d’un travailleur au salaire minimum au Canada est de 29 250 dollars canadiens. Les infirmières autorisées gagnent en moyenne 72 783,75 dollars canadiens par an. Le coût de fonctionnement d’une clinique privée ne comptant qu’un(e) employé(e) au Canada est en moyenne de 102 500 dollars canadiens par an. Par conséquent, l’embauche d’un chaperon à temps plein augmenterait les dépenses de la clinique d’environ 49 % par an, soit un total approximatif de 153 517 dollars canadiens par an. Dans le cas d’une embauche à temps partiel, le coût annuel d’un chaperon serait d’environ 10 203 dollars canadiens pour chaque jour/semaine d’emploi. **Conclusion:** En termes de considérations financières, l’embauche d’un chaperon peut multiplier les dépenses de la clinique par 1,5. Les constatations de cette étude fournissent une référence importante aux médecins et peut les aider dans leur décision d’embaucher des chaperons en pratique clinique.

## Introduction

For the past several years, medical associations and medicolegal bodies have been increasingly advocating for the use of chaperones in clinical practice.^1–3^ Chaperones are individuals who can be called upon to provide witness during physical examinations of a sensitive nature.^1,4–6^ Such examinations include those of the breast (for breast reductions, augmentations, reconstructions, and mastopexies) and of the pelvis. They can also include examinations related to body contouring procedures and of patients with mental health concerns. As witnesses, chaperones provide protection for both the patients and physicians: they can provide protection for the patients from abuse or exploitation by physicians, and for physicians from false accusations of assault or sexually inappropriate behavior by the patients. They can also provide reassurance for patients during routine parts of the examination.^4,5,7–9^

Most commonly, nurses take on the role of chaperones in clinical practice.^5,6,10^ Chaperones can also be other authorized members of the clinical staff, such as midwives or physician assistants.^5,8,11,12^ In some cases, they may be the patient's partner or a support person of their choosing, such as a family member, friend, or support worker.^5,8^ In academic settings, residents can take on the role of a chaperone; unless they themselves are alone with the patient. Ultimately, the need for a chaperone is higher in private practice as compared to academic practice. Although chaperones are used in various clinic practices, such as those of family doctors,^6^ they are of particular importance in practices where sensitive physical examinations are frequently required, such as Obstetrics and Gynecology and Plastic Surgery clinics.^5^

Routine chaperone use is still quite limited worldwide. One of the barriers to universal chaperone use is scarcity of available personnel, which makes it difficult to have chaperones present for every sensitive examination.^4,5,8,13,14^ For instance, Cuccolo et al indicated that 74.7% of surveyed clinicians did not use chaperones due to limited availability. Another commonly cited barrier is the financial implications of hiring a chaperone.^8,14,15^ To mitigate the cost, physicians may rely on nonmedical employees such as receptionists to act as chaperones^6,10^; however, their availability may be limited by their other office responsibilities.

Ultimately, there is a noticeable paucity of publications and available information regarding the cost of hiring an individual to solely act as a chaperone. The objective of this study is to examine the financial aspects of hiring a dedicated chaperone for regular use in clinical practice.

## Materials and Methods

The cost of hiring a dedicated chaperone in a private clinic was examined by analyzing the range of possible salary rates for clinic staff who can act as a chaperone in Canada: from a minimum-wage earning staff member to the highest earning hired clinic staff member, a Registered Nurse (RN). These salaries were determined based on information available on the official Government of Canada website.^16^ To understand the financial implications for physicians of hiring a chaperone, the range of these salaries was considered in the context of general expenses involved in operating a clinic, including: staffing expenses; electronic medical records; medical equipment; office space lease; and insurance. Costs were calculated in Canadian Dollars (CAD). Obstetrics and Gynecology and a Plastic Surgery urban community clinics in Canada were consulted regarding their operating costs.

## Results

The results of this study are summarized in Table 1. In Canada, the wage of a Registered Nurse can range from $26.28 to $48.37 CAD per hour (not limited to private clinic practice).^16^ Taking an approximate mean wage of $37.33 per hour, and full-time employment of 1950 hours of service each year without accounting for unpaid time,^17^ the annual mean nursing salary is $72,783.75 CAD.

**Table 1.** Financial Implications of Hiring a Dedicated Clinical Chaperone.

	Hourly wage (CAD/h)	Annual wage (CAD)^a^	Annual cost of medical practice^b^	Increased mean annual cost of medical practice with a full-time chaperone	Percent mean increase in annual cost of medical practice with a full-time chaperone
Nurse	$26.28-48.37 (mean = $37.33)	$51,246-94,321.5 (mean = $72,783.75)	$75,000-130,000 (mean = $102,500)	$175,283.75	71.01%
Minimum wage employee	$15	$29,250		$131,750	28.54%

^a^ Based on 1950 h of service each year.

^b^ Medical practice comprising of a physician and one other clinical staff member (usually a secretary).

As of the end of 2021, the minimum wage in Canada is $15 per hour.^18^ With the same hours of service each year as nurses, the annual cost for hiring a full-time chaperone at a minimum wage is $29,250 CAD.

The cost of maintaining a medical practice comprising of a physician and one other clinical staff member (usually a secretary) is generally between $75,000 and $130,000 CAD per year.^19, personal communication^ With a mean cost of $102,500 CAD to operate a practice, hiring a full-time chaperone with the salary of a nurse would increase clinic expenses for the physician by approximately 71.01%. Conversely, hiring a full-time chaperone with a minimum wage salary would increase costs by approximately 28.54%. Thus, hiring a chaperone could increase clinic expenses by approximately a mean of 49.75% ($51,017 CAD) per year, thus bringing total clinic expenses to approximately $153,516.88 CAD per year.

Considering that full-time employment of 1950 hours implies a 5-day workweek, the annual employment of a chaperone for 1 day/week will cost approximately $5850 to $14,556.7 CAD (mean = $10,203 CAD). As a simplified rule of thumb, the annual cost of hiring a chaperone is approximately $10,000 CAD for each day/week of employment.

## Discussion

With increasing reports of sexual misconduct,^20,21^ it is paramount to ensure patient safety and physician accountability in clinical practice. At the same time, it is also important to ensure that physicians are protected against false accusations by patients.^4,5,7–9^ Although it is critical for chaperones to be available in any clinical practice where sensitive examinations are required, their presence is perhaps more so important in practices such as Obstetrics and Gynecology and Plastic Surgery, which frequently rely on pelvic or breast examinations for diagnosis and management.

Presently, there is no clinical role designated solely as “chaperone”. Although nurses are commonly used as chaperones,^5,6,10^ they can have many other roles in clinical practice, such as conducting initial intake of patients, taking their vital signs and measurements, and administering vaccinations. Therefore, while relying on a nurse as a chaperone would be sensical in a practice where sensitive examinations do not comprise most of the appointments, it would be inefficient to hire a nurse solely for the task, considering their extensive skillset.

As highlighted by the Canadian Medical Protective Association, while it is possible to hire an individual at minimum wage to act as a chaperone, it is preferable that they have training to be familiar with the physical examinations in order to confirm that they were conducted appropriately.^1^ Medical office chaperone training is offered by some Canadian colleges, such as the College of Physicians & Surgeons of Alberta, with the cost of the course being under $500 CAD.^11^ In some instances, patients can bring in their partner or another support person of their choosing to act as a chaperone. However, a patient support person may not be available in all cases, and with the COVID-19 pandemic, nonessential care partners are not allowed to be present in some settings due to social distancing restrictions. A patient's partner also lacks the necessary training, understanding of, and impartiality to the examination.

For a practicing physician, it is important to know the financial implications of hiring a dedicated staff member to act as a chaperone. The financial considerations of hiring a dedicated chaperone for clinical practice would be between the cost of hiring an individual at minimal wage and that of a nursing salary. Hiring a full-time chaperone for clinical practice may cost anywhere from $29,250 CAD to $72,783.75 CAD. Based on the results of this study, this can increase the costs of a clinic comprising of a physician and one other clinic staff member by approximately 49.75% per year, bringing the average annual cost from $102,500 CAD to $153,517 CAD per year. This significant cost highlights why many private physician practices prefer to operate with minimal additional clinic staff and may hire only a secretary for administrative purposes. Of course, hiring a part-time chaperone would have a fraction of this cost; the annual cost of hiring a chaperone would be approximately $10,000 CAD for each day/week of employment.

There are several methods that physicians can use to reduce the cost of hiring a chaperone. This includes operating in a shared practice with other physicians, in which the cost of a chaperone can be split among other members. Additionally, physicians can consider booking patients requiring sensitive examinations in adjacent time blocks. For example, patients requiring pelvic or breast examinations can be booked in the morning, while patients requiring less sensitive examinations can be booked in the afternoon. This will limit the time that a chaperone will need to be present in the clinic.

The additional cost of hiring a chaperone may affect male physicians to a greater extent than female physicians. Several studies have indicated that approximately 29% or less of female patients wished for a chaperone when being examined by female physicians, while up to 32% to 75% of female patients wished to have a chaperone present when being examined by a male practitioner.^13,22,23^ Moreover, multiple studies have shown that male physicians are more likely to use chaperones than their female counterparts.^5,6,9,24^ As Newton et al notes, such findings suggest that chaperones should be routinely offered to female patients being examined by male practitioners.^9^

This research is primarily limited by the fact that there can be variability in the salaries of nursing clinic staff based on their location of practice and clinical experience. This makes it difficult to provide more specific values when trying to calculate the possible range for chaperone salaries. Similarly, the study did not take into account any particulars of employment, such as time for unpaid leave, which may differ between individuals and practices. Additionally, while this research study did consult with community physicians regarding annual costs, expenses for operating their clinics can vary significantly based on the location of the practice (ie, hospital vs community; urban vs rural), how many staff they presently employ, and whether they are working as part of a group of physicians that can split the costs. Therefore, the financial implications of hiring a dedicated chaperone can vary between practices.

## Conclusion

Governing and legal medical bodies are increasingly encouraging the use of chaperones for sensitive physical examinations of patients in clinical practice. Although nurses are commonly used for this role, it may not always be the most efficient use of their capabilities and resources in a busy private practice requiring frequent sensitive exams. Using a minimum wage worker as a chaperone is possible; however, they must receive certified training to be most effective. Based on the results of this study, hiring a dedicated chaperone may amount to an approximate 49.75% increase in annual operating clinic expenses for a clinic already comprising of a physician and one other clinic staff member. Employing a chaperone part-time, based on the actual needs of a physician's practice, will result in correspondingly adjusted costs (approximately $10,000 annual cost for each day/week of employment).

The findings of this study provide an important reference for physicians in terms of the financial considerations of hiring a chaperone. It can thus encourage the greater utilization of chaperones in clinical practice by physicians, which would help alleviate the need to rely on nursing or untrained staff for sensitive examinations. Finally, considering the associated costs of hiring a chaperone, it is worth reflecting on whether there should be increased financial billing and payment adjustments for physicians to accommodate the increasing urging by medicolegal regulators for routine implementation of chaperones.
